# An Algorithm for Timely Transmission of Solicitation Messages in RPL for Energy-Efficient Node Mobility

**DOI:** 10.3390/s17040899

**Published:** 2017-04-19

**Authors:** Jihong Park, Ki-Hyung Kim, Kangseok Kim

**Affiliations:** 1 Department of Computer Engineering, Graduate School of Ajou University, Suwon 16499, Korea; nicehong@ajou.ac.kr; 2 Department of Cyber Security, Ajou University, Suwon 16499, Korea; kkim86@ajou.ac.kr

**Keywords:** low power and lossy networks, RPL, mobility, energy efficiency, solicitation transmission

## Abstract

The IPv6 Routing Protocol for Low Power and Lossy Networks (RPL) was proposed for various applications of IPv6 low power wireless networks. While RPL supports various routing metrics and is designed to be suitable for wireless sensor network environments, it does not consider the mobility of nodes. Therefore, there is a need for a method that is energy efficient and that provides stable and reliable data transmission by considering the mobility of nodes in RPL networks. This paper proposes an algorithm to support node mobility in RPL in an energy-efficient manner and describes its operating principle based on different scenarios. The proposed algorithm supports the mobility of nodes by dynamically adjusting the transmission interval of the messages that request the route based on the speed and direction of the motion of mobile nodes, as well as the costs between neighboring nodes. The performance of the proposed algorithm and previous algorithms for supporting node mobility were examined experimentally. From the experiment, it was observed that the proposed algorithm requires fewer messages per unit time for selecting a new parent node following the movement of a mobile node. Since fewer messages are used to select a parent node, the energy consumption is also less than that of previous algorithms.

## 1. Introduction

RPL is a routing protocol devised for low power and lossy networks (LLN) that consist of resource-limited devices. As a routing protocol that operates among IoT (Internet of Things) devices, RPL was designed to be suitable for wireless sensor network environments, and it supports various routing metrics to accommodate the requirements of various applications [1]. The RPL forms a wireless sensor network using a directed acyclic graph (DAG) topology so that the cost of every node reaching the LLN border router is minimized. Moreover, a trickle algorithm is used to maintain consistency among the nodes that comprise the network [1,2]. While such a design is optimized for IoT services where the network topology does not vary, it is difficult to maintain the routing paths in a stable manner in an IoT service environment where the devices move frequently [3,4]. Therefore, considering that mobility is an important property of mobile devices in an IoT environment, there is a need for a mechanism that is energy efficient and that provides for stable and reliable data transmission.

Mobility support is a fundamental issue in RPL. Previous works on mobility support in RPL mostly aimed to achieve rapid detection of the movement of mobile nodes and the provision of better links through an efficient selection of parent nodes. In other words, mobility support in RPL is directly related to a fast search for appropriate nodes to become the parent node when a mobile node makes a movement. Moreover, previous works on mobility support in RPL focused on improving the trickle algorithm used in RPL [5,6,7,8,9,10,11]. From a methodological perspective, previous works mostly used algorithms that increase or decrease the time interval for the selection of parent nodes by multiplying or dividing a predefined interval by a certain integer value. This was based on the respective methods proposed in each work in order to support the mobility of the nodes. However, mobile nodes are objects that move in a random manner in a network. Therefore, the methods proposed by previous works can lead to losses in the intervals during which the mobile nodes select the parent node in an RPL network. This loss may cause the increase of the packet loss rate and energy consumption while the information is delivered. Hence, considering these characteristics of mobile nodes, the interval during that a mobile node selects a parent node must be allocated dynamically based on the speed and direction of the movement of the node.

This paper proposes an algorithm for the timely transmission of solicitation messages for node mobility support in IoT environments that require the movement of the nodes or things. The proposed algorithm provides mobility support by dynamically adjusting the transmission period of the messages for selecting the parent node. This is based on the speed and direction of the movement of a mobile node.

The rest of this paper is organized as follows. Section 2 explains the basic concepts of RPL and the trickle algorithm related to our study and brings the problems occurred when nodes have mobility in RPL. To solve these problems, Section 3 proposes an algorithm that supports mobility considering the velocity and direction of the node in RPL. Section 4 presents the simulation environment. Section 5 compares the proposed algorithm with the existing algorithms based on the simulation results, and the performance is evaluated. Finally, this paper is concluded with future research directions in Section 6.

## 2. Motivation and Related Works

The RPL standard [1] defines the generation and management of destination-oriented DAG (DODAG), which is a tree-based routing topology in IPv6 wireless sensor networks. Moreover, it supports multipoint-to-point, point-to-multipoint and point-to-point data transmission based on DODAG. RPL builds an entire topology graph by using three different ICMPv6-based control messages: DIO (DODAG information object), DAO (DODAG destination advertisement object) and DIS (DODAG information solicitation).

In RPL, the creation and management of a DODAG starting from the DODAG root are achieved through the transmission of DIO messages. The DODAG root creates a DODAG and spreads the DODAG information to the nodes in the network through the transmission of DIO messages. Nodes that receive a DIO message participate in the DODAG and generate an upward path toward the DODAG root. The routers in the network that are capable of broadcasting allow for the other nodes to participate in the DODAG by transmitting DIO messages. The nodes that are currently participating in DODAG generate a downward path using DAO messages. DIS is used to solicit DIO messages from router nodes in RPL. DIS may be used to probe neighbor nodes in adjacent DODAGs. After receiving the DIO messages, the new node then selects one or more parent nodes using the objective function (OF).

The trickle algorithm in RPL is used to maintain consistency in a network by using control messages [2]. The trickle algorithm reduces energy consumption by avoiding duplicate messages between nodes. For this purpose, the trickle algorithm exchanges information with network nodes in a highly robust, energy efficient, simple and scalable manner. The trickle algorithm rapidly detects changes in the network topology and adjusts the network to a consistent state using only a few control messages per hour after the network topology is stabilized. The simple suppression mechanism and transmission point selection used in the trickle algorithm are based on simple rules rather than a complicated synchronization scheme between nodes. Owing to these characteristics, even with a linear increase in the number of nodes, the overall traffic increases logarithmically.

A trickle timer governs the transmission of control messages for adjusting the network to a consistent state. The timer controls the inconsistencies in an RPL network and prevents duplicate transmissions of DIO messages. If an RPL network is in an inconsistent state, the interval of the trickle timer becomes shorter, and control messages are transmitted more frequently in order to stabilize the network. By contrast, if an RPL network is in a consistent state, the number of control message transmissions is reduced by increasing the trickle timer interval in order to reduce the overhead in the network incurred owing to control messages.

If there is a node that moves frequently in an RPL network, a route that is suitable for movement must be selected for this node [4,10]. For example, when the network is stable, the DIO messages spread slowly owing to the trickle timer. In this case, if a mobile node moves to another location, it may not be able to participate in the network owing to the slow spread of DIO messages. Indeed, this node may request a DIO message by transmitting a DIS message to a neighboring router node from the new location. However, such a mechanism diverges from the RPL standard. As such, the node must wait until a DIO message is received from the neighboring router nodes or the trickle timer must be reconfigured in an artificial manner. Therefore, appropriate scenarios must be established for mobility support in RPL [12]. Based on this, the trickle algorithm needs to be modified.

There have been several studies to improve the trickle algorithm with the support of node mobility in RPL. Ghaleb et al. [6] proposed an enhanced version of the trickle algorithm, which tried to solve the problem of increased latency incurred from a listen-only period in the trickle algorithm. The listen-only period in the network increases the convergence time. Korbi et al. [10] described the necessary elements for the node mobility support in RPL, and an RPL protocol is proposed that is optimized for upward routing from a mobile node to a sink node in a fixed network. The algorithm in the paper dynamically adjusts the DIS message interval to enable faster detection of network topology changes due to node movement.

The algorithms of [6,10] are to adjust the trickle timer, which could support node mobility, but there are a few disadvantages. In [6], the algorithm untimely set the time interval for control messages in order to solve the short-listen problem. When inconsistent information is received, a control message is transmitted due to the redundancy constant. This method can cause frequent transmission and reception of control messages. In addition, the methods in [6,10] adjust the trickle timer by arithmetically operating a constant value without considering the speed and the moving distance of the mobile node. Therefore, if two algorithms are applied to the LLN with a mobile node having a variable speed, packet loss and energy consumption may be increased when the mobile node selects a parent node and transmits information.

In this paper, we propose an algorithm that dynamically allocates the time interval of selecting the parent node by considering the speed and the moving distance of a mobile node. We also present a scenario for selecting parent nodes considering the situation of moving based on the dynamic time interval.

## 3. Proposed Algorithm for Energy-Efficient Node Mobility

In this section, we propose an algorithm to support node mobility in RPL and describe its operating processes based on several scenarios. The operation procedure of the proposed algorithm is described in Section 3.1. Section 3.2 explains how to calculate the time interval occurring as a mobile node selects a parent node based on the moving speed and distance of the mobile node. Section 3.3 describes scenarios of selecting the parent node of a mobile node.

### 3.1. Procedure of the Proposed Algorithm

The proposed algorithm is identical to the RPL standard in that DIS and DIO messages are used to search for routes, and DAO messages are used to select a path. However, the proposed algorithm supports the mobility of nodes by dynamically adjusting the DIS message transmission period based on Doppler frequency and signal strength between a mobile node and its neighboring router nodes.

**Figure 1:**
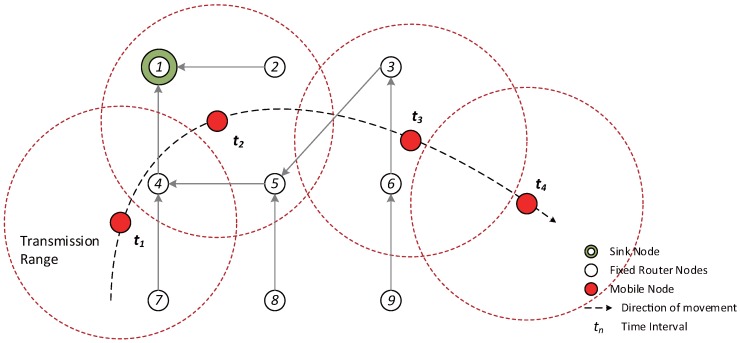
An example of the movement of a mobile node in RPL with time.

Moreover, the proposed algorithm considers a network environment where both fixed and mobile nodes exist, and the mobile nodes are assumed to operate as leaf nodes without broadcasting owing to limitations in power supply. Figure 1 shows an example of the movement of a mobile node in an RPL network with increasing time. At each step, the mobile node selects the most suitable parent node after moving to the new location.

- $t_{1}$: The mobile node participates in LLN for the first time, selects the parent and forms DODAG.
- $t_{2}$: The information of the previous parent node is renewed while the mobile node is moving.
- $t_{3}$: The information of the previous parent node does not exist at the new location.
- $t_{4}$: The mobile node leaves the LLN.

**Figure 2:**
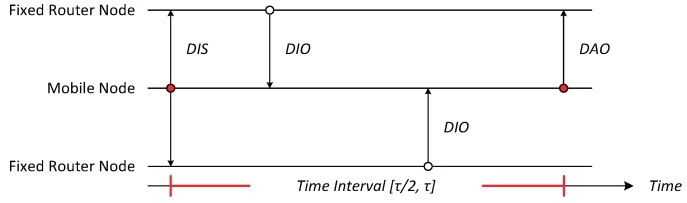
An example operation of the proposed algorithm.

**Figure 3:**
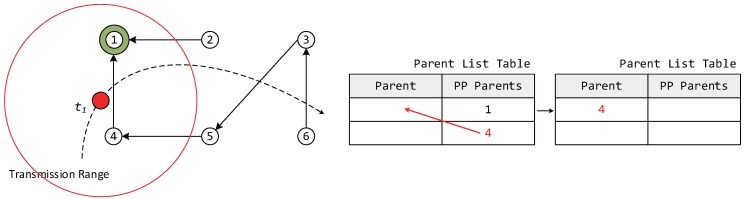
An example of selecting the parent of a mobile node from preferred parents.

When a mobile node attempts to participate in a network by configuring the DODAG information, a DIS message is transmitted to neighboring router nodes to find its new parent node. As soon as the neighboring router nodes receive the DIS message, the nodes transmit a DIO message to the mobile node as shown in Figure 2. Upon receiving the DIO message, the mobile node stores both a selected node ID and an OF value for choosing the parent node in a parent list table similar to the one shown in Figure 3. The entries in a parent list table can be classified as the parent and preferred parents (PP). Once the mobile node receives a DIO message, the information of neighboring router nodes is stored as PPs in the parent list table. Once the interval for selecting a new parent node when mobile node moves from one location to another expires, the router node with the highest OF value among the PPs is selected as a new parent, and the entry of the parent columns is updated with the value (new parent).

Then, the mobile node extracts the values of the Doppler frequency and signal strength from the DIO message. The Doppler frequency is used to measure the moving speed of a mobile node, and the signal strength is calculated to measure the distance between a mobile node and router nodes [13,14,15,16]. Therefore, a time interval can be determined dynamically from the values. Then, the mobile node sends a DAO message to a selected router node based on the time interval. The selected router node will become a new parent node. The time interval is initialized after the selection.

The proposed algorithm, which is executed on the mobile node, is composed of five phases as follows. The pseudocode for the proposed algorithm is described in the table in Algorithm 1.

1. Initialization phase: While the proposed algorithm is similar to the trickle algorithm in that $I_{min}$ and $I_{max}$ are set, the proposed algorithm does not have a redundancy constant *k*. Instead, it has a parent list table. When the algorithm is first executed, $I_{MTP}$ is set as $I_{min}$, and the parent list table and the current time are initialized. The $I_{MTP}$ represents an interval where MTP means migration time predictable. The interval $I_{MTP}$ is described in more detail in Section 3.2.3. The time information possessed by a mobile node is based on the time measured using the internal clock of the device. Moreover, because the information is used independently in the network, there is no need to synchronize the time of the entire network.
2. Receiving information of neighboring router nodes: When the algorithm is started, DIS messages are transmitted to the neighboring router nodes, and DIO messages required for parent selection are received from the neighboring nodes until $I_{MTP}$ expires.
3. Scenario-based parent selection: Afterward, if the current time reaches $I_{MTP}$, a parent node is selected based on the scenarios presented in Section 3.3.
4. Adjusting the time interval: A DAO message is sent to the router node selected as the parent, and the interval used for the selection of the next parent is dynamically adjusted based on Equation (7) described in Section 3.2.3.
5. Reinitialization of parameters: Next, the current time of the mobile node and the PP attributes in the parent list table are initialized. If there are no values for the PP attributes in the parent list table when the current time reaches $I_{MTP}$, all values necessary for the mobile node to select the parent node are reset to the values when the algorithm was first executed.

**Algorithm 1** Proposed algorithm1:/*Theinputandoutputoftheproposedalgorithm*/2:INPUT : Information of router nodes within one hop from a mobile node3:OUTPUT : Parent node ID4: 5:/*Macrodefinition*/6:define $\;I_{min}$7:define $\;I_{max}$8:struct nTable { nID, OF }9: 10:/*1.Initializationphase*/11:$I_{MTP}$ = $I_{min}$, cTime = 012:struct nTable$\;nTable_{old}\left[\;\right]$13:struct nTable$\;nTable_{new}\left[\;\right]$14: 15:/*2.Receivinginformationofneighboringrouternodes*/16:**loop**17: **if** cTime = 0 **then**18:  send DIS to Router Nodes by one-hop broadcasting19: **end if**20: 21: **while** cTime ≤$I_{MTP}$ **do**22:  **if** received DIO **then**23:   $nTable_{new}\left[i\right].nID$←router_nodeID24:   $nTable_{new}\left[i\right].OF$←OF25:  **end if**26: **end while**27: 28: /*3.Scenariobasedparentselection*/29: **if** cTime = $I_{MTP}$ **then**30:  **if** $nTable_{new}$≠ NULL **then**31:   **if** $nTable_{old}$ = NULL **then**32:    $nTable_{old}.nID$←router_nodeID selected by highest OF in $nTable_{new}$33:   **else if** parent node ID in $nTable_{old}$ = router_nodeID in $nTable_{new}$ **then**34:    **if** parent node OF in $nTable_{old}$ < Threshold to request signal level **then**35:     $nTable_{old}.nID$←router_nodeID selected by highest OF in $nTable_{new}$36:   **end if**37:  **else**38:    $nTable_{old}.nID$← parent node ID in $nTable_{old}$39:   **end if**40: 41:   send DAO to a selected router node42: 43:   /*4.Adjustingthetimeinterval*/44:   $I_{MTP}$ = xwhereτ/2≤x<τ45: 46:   **if** $I_{MTP}$ > $I_{max}$ **then**47:    $I_{MTP}$ = $I_{max}$48:   **else if** $I_{MTP}$ < $I_{min}$ **then**49:    $I_{MTP}$ = $I_{min}$50:   **end if**51: 52:   **else**53:    $I_{MTP}$ = $I_{min}$, $\;nTable_{old}$ = NULL54:   **end if**55: 56:   /*5.Reinitializationofparameters*/57:   $nTable_{new}$ = NULL, cTime = 058: 59: **end if**60:**end loop**


### 3.2. A Time Interval Calculated According to the Moving Speed and Distance of a Mobile Node

#### 3.2.1. Velocity Estimation of the Mobile Node

As a mobile node moves from one location to another, a frequency difference occurs in the received signal owing to the Doppler effect. This frequency difference is referred to as Doppler frequency. The maximum Doppler frequency in the received signal is proportional to the speed of motion of the mobile node. The relative speed of the mobile node is related to the signal sent from the neighboring router node. The signal can be used to find the speed and direction of the mobile node [13,16]

If a neighboring router node is in motion relative to a mobile node, the frequency of the signal received by the router node is different from that of source signal. If a mobile node moves closer to a router node, the received signal has a higher frequency than the transmitted signal. When a mobile node moves farther away from a router node, the received frequency decreases.

If the speed of motion of a mobile node is $V_{m}$, the angle between the mobile node and the parent node is θ, the speed of the electromagnetic (EM) wave is *c*, $f_{d}$ is the Doppler frequency, the received frequency is $f_{r}$ and the frequency transmitted from router nodes is $f_{t}$, then the Doppler frequency is given by Equation (1):

(1) $$f_{d}=f_{r}-f_{t}=V_{m}\cdot \cos\theta \cdot f_{t}/c$$

To find the dynamic time interval proposed in this paper, we first need to know the speed of the mobile node. Using Equation (1), the speed of a mobile node can be estimated as shown in Equation (2):

(2) $$V_{m}=f_{d}\cdot c/\cos\theta \cdot f_{t}$$

#### 3.2.2. Estimation of Distance to Escape the Transmission Range of a Parent Node

**Figure 4:**
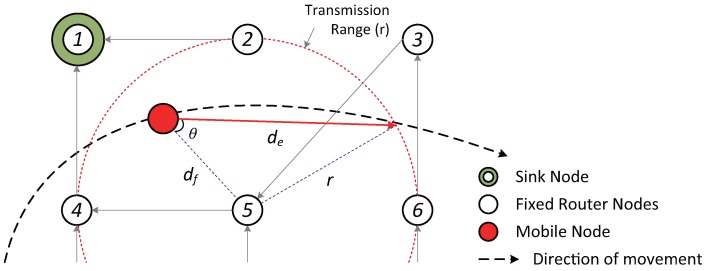
Estimation of the distance required to escape the transmission range of a parent node.

As shown in Figure 4, when a mobile node approaches or recedes from a parent node, the mobile node must travel a distance (e.g., $d_{e}$ in the figure), required for the mobile node to escape the transmission range of the parent node. Then, the mobile node is allowed to find the next parent node rapidly to form another DODAG by defining the interval to be the time taken to travel the distance to escape from the transmission range of the parent node. In order to get the distance $d_{e}$, the values of θ and $d_{f}$ are required where θ and $d_{f}$ are the angle and the distance between the mobile node and the parent node (node Number 5 in the figure) respectively. In this paper, we assume that the direction of movement can be measured by applying such methods as sideband filtering, offset carrier demodulation, in-phase/quadrature demodulation, etc., to the Doppler frequency sent from the router node [16,17]. Therefore, the value of θ to get the direction can be obtained from Equation (1), and $d_{f}$ can be estimated from the received signal strength indicator (RSSI) value.

The current method estimates the distance based on the fact that the RSSI varies with distance. By transmitting a signal of fixed strength from the router node, the distance can be estimated using the strength of the attenuated signal received by the mobile node. Therefore, the transmission loss (*L*) in the transmitted signal is calculated as the difference between the two signals and can be found using the Friis free space propagation model [18,19,20].

(3) $$L=20\log_{10}\cdot 4\pi d_{f}/\lambda \;\left[dB\right]$$

In Equation (3), λ represents the wavelength of the EM wave. Equation (3) can be rearranged as Equation (4) in order to estimate the distance between the mobile and router nodes.

(4) $$d_{f}=\lambda /4\pi \cdot 10^{L/20}=c/4\pi f_{r}\cdot 10^{L/20}$$

Another factor required to calculate the dynamic time interval is to obtain $d_{e}$ shown in Figure 4. Since the line segments $d_{f}$, *r* and the angle θ are known, the $d_{e}$ can be obtained by applying the second law of cosines where *r* is the transmission range of the router node. By calculating the Equation (5), the distance value can be obtained.

(5) $$d_{e}=d_{f}\cdot \cos\theta +\sqrt{(d_{f}\cdot \cos\theta {)}^{2}+r^{2}-{d_{f}}^{2}}$$

#### 3.2.3. Calculation of the Interval in the Proposed Algorithm

In the proposed algorithm, we define the interval as the time before a mobile node escapes the transmission range of the parent node. The interval is inversely proportional to the speed of the mobile node and proportional to the distance required for the mobile node to escape the transmission range of the parent node, $d_{e}$. In other words, the proposed algorithm dynamically adjusts the DIS transmission period based on the speed and distance of the mobile node. However, owing to the random nature of the speed and direction of the mobile node, there can be a time error in escaping the transmission range of the parent node. To overcome this, the proposed algorithm sets the range of intervals as [τ/2, τ], and the interval is randomly selected within this range. The detected interval of length τ can be calculated using Equation (6) based on Equations (2) and (5), and the interval can be obtained using Equation (7):

(6) $$\tau =d_{e}/V_{m}$$

(7) $$I_{MTP}=x,\;\;\mathrm{where}\;x\in \left[\tau /2,\tau \right]$$

Here, *x* is a value randomly selected from [τ/2, τ], and $I_{MTP}$ represents the interval where MTP means migration time predictable.

### 3.3. Scenarios Based on Mobile Node Movement

#### 3.3.1. Case of a Mobile Node Participating in LLN for the First Time, Selecting the Parent and Forming a DODAG

**Figure 5:**
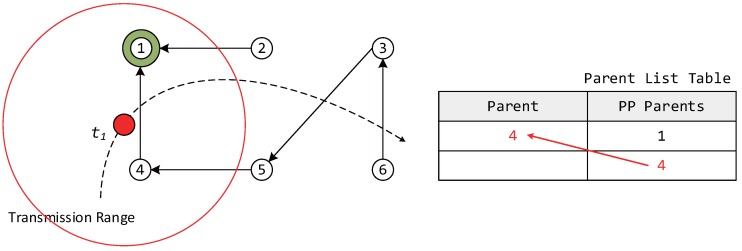
Update state in the parent list table when mobile node first participates in low power and lossy networks (LLN).

In this section, we describe the case of a mobile node participating in an LLN for the first time. In Figure 5, because the mobile node does not currently have a parent node selected, it must quickly select a parent node, form a DODAG, and participate in the network. Therefore, the initial value of $I_{MTP}$ is selected to be $I_{min}$ in order to select the parent node rapidly. The mobile node receives DIO messages from neighboring router nodes for a duration of $I_{min}$ after transmitting a DIS message. At this point, the ID and OF information of the neighboring router nodes is added to the PP column of the parent list table.

The mobile node can measure the Doppler frequency upon receiving a DIO message. The speed of the mobile node is estimated based on Equation (2) and is used in dynamically adjusting $I_{MTP}$ after the use of $I_{min}$. After $I_{min}$, the mobile node selects the node with the highest OF value among the neighboring router nodes with different IDs based on its PP information and transmits a DAO message to the newly-selected parent node, forming a DODAG. After the selection of the parent node, only the information of the selected parent is moved to the parent column, and this information is used in later steps. After selecting the parent node, $I_{MTP}$ is dynamically allocated based on the speed of the mobile node and $d_{e}$ in Figure 4, using Equation (7).

#### 3.3.2. Case of Parent Node Information Being Renewed during the Motion of the Mobile Node

In this section, we describe the case where the parent node information is renewed while the mobile node is moving. The same process as described in Section 3.3.1 is used to receive the information of neighboring router nodes and to store the information in the PP column of the parent list table upon receiving DIO messages followed by DIS message transmission. Afterward, the OF values of a PP node selected from the parent list table and the previous parent node are compared.

During this process, if there is a router node with the same ID as the parent node selected at $t_{n-1}$, then the OF value of this node is examined. If the OF value of this node is higher than a threshold, then the router node selected at $t_{n-1}$ is left as the parent node in the next step. If not, the router node with the highest OF value in the PP column of the parent list table is selected as the new parent node. Here, the threshold represents the signal strength that allows for smooth communication between the mobile and parent nodes.

**Figure 6:**
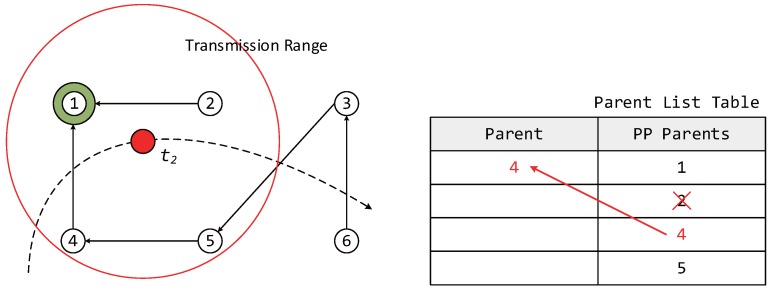
Update state in the parent list table when the parent node information is renewed during the movement of the mobile node.

In the example shown in Figure 6, while the router node ID with the greatest OF value in the PP column is 2, because the OF value of the previous parent node is higher than the threshold, router node ID 4 continues to be used as the parent node. The method for the dynamic allocation of $I_{MTP}$ after parent node selection is that of Section 3.3.1.

#### 3.3.3. Case Where the Information of the Previous Parent Node Is Not Found at the New Location

**Figure 7:**
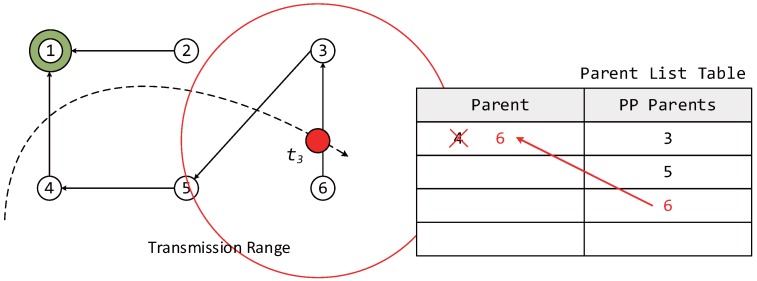
Update state in the parent list table when the information of the previous parent node is not found at the new location.

In this section, we describe the case where the information of the previous parent node is not found at the new location of the mobile node. The mobile node stores the information of neighboring router nodes received at $t_{n}$ in the PP column of the parent list table. This process is as described in Section 3.3.1. During this process, the information of the parent node selected at $t_{n-1}$ is compared with that of the neighboring router nodes received at $t_{n}$, as described in Section 3.3.2. If the mobile node cannot find router node information that is identical to the information of the parent node selected at $t_{n-1}$, the neighboring router node with the highest OF value received at $t_{n}$ is selected as the parent node. Figure 7 describes the case where the parent node selected at time $t_{2}$ does not exist among the neighboring router nodes received at $t_{3}$. Similarly, $I_{MTP}$ is dynamically allocated after selecting the parent node to support node mobility.

#### 3.3.4. Case Where a Mobile Node Escapes the LLN

This section describes the case where the mobile node escapes the LLN while moving freely. After transmitting a DIS message, the mobile node receives a DIO message for the duration of $I_{MTP}$ allocated at $t_{n-1}$ in order to collect the information of neighboring router nodes. However, if the mobile node escapes the LLN while moving, DIO messages cannot be received even after waiting for $I_{MTP}$ after DIS message transmission because there are no router nodes nearby. Therefore, there is no router node information in the PP column of the parent list table of the mobile node when $I_{MTP}$ expires. If there is no PP information in the parent list table at the end of $I_{MTP}$, the parent list table of the mobile node is reinitialized because there is no information on the parent nodes that can be selected.

The absence of information in the parent list table of a mobile node implies that there are no router nodes nearby. Therefore, the mobile node must collect the information of the neighboring router nodes as soon as possible and thus select a parent node. During this process, $I_{MTP}$ is set as $I_{min}$ regardless of the speed of the mobile node. This is done in order to allow for a fast re-participation in the LLN.

## 4. Simulation Environment

### 4.1. Parameters and Network Topology for Simulation

The proposed algorithm finds the time interval used by the mobile nodes to select the parent node in a dynamic manner. An experiment was conducted using MATLAB to compare and evaluate the proposed algorithm, the original trickle algorithm [2], the E-trickle (Enhanced trickle) algorithm [6] and the ME-RPL (Mobility Enhanced RPL) [10], which are used to adjust the network to a consistent state. The parameters used for the simulation are listed in Table 1. The packet size was set as 32 bytes including the RPL header, DODAGID and payload. A random waypoint model was used to simulate the motion of the mobile node [21]. In the random waypoint model, the average human walking speed was set as the minimum and the average running speed as the maximum. The speed of the mobile node was selected randomly within a range defined by the minimum and maximum. The minimum interval for the mobile node was set as $2^{12}$ ms, and the maximum interval was set as $2^{20}$ ms, as defined by Contiki [22]. The interval used for selecting the parent node was determined based on each algorithm. During the experiment, the *k* value must be configured with a default value in order to exclude the duplicate information from neighboring router nodes. Therefore, in this paper, a *k* value of two was used for the original trickle algorithm and the E-trickle algorithm during the experiment.

**Figure 8:**
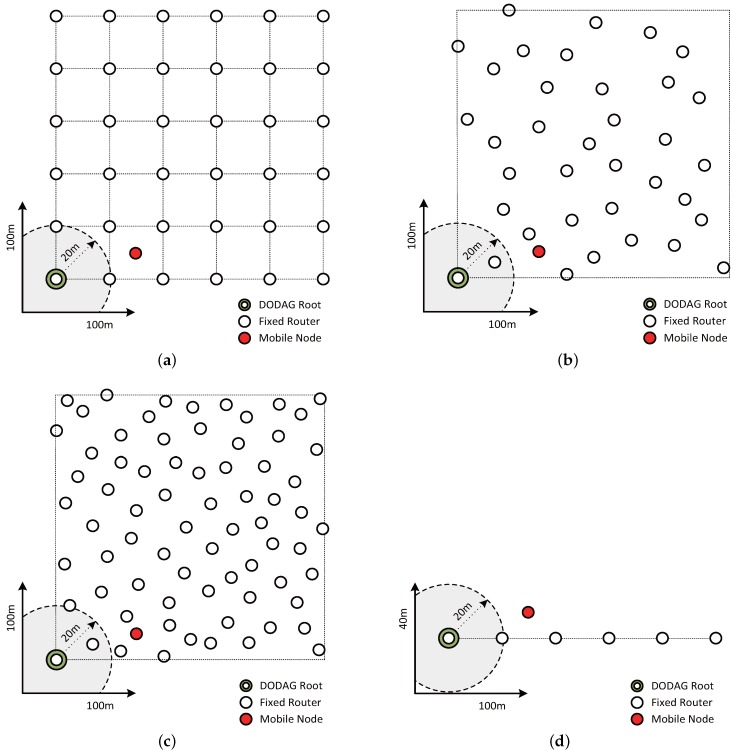
Network topology for simulation. (**a**) Grid topology with 36 router nodes; (**b**) Random topology with 36 router nodes; (**c**) Random topology with 72 router nodes; (**d**) Linear topology with 6 router nodes.

In order to compare the performance of our proposed algorithm with existing methods, three criteria were evaluated using different network topologies, as shown in Figure 8. Each experiment was conducted for 5000 s. The three criteria are the number of DIS message transmissions, the packet loss rate of the transmitted messages and the energy consumed during the transmission and reception of messages.

To establish a grid network topology as shown in Figure 8a, 36 fixed router nodes were placed at a certain interval. Moreover, in order to perform an experiment in a random topology network that was similar to most actual environments, 36 and 72 fixed router nodes placed at random intervals were used, as shown in Figure 8b,c, respectively. The last network topology used for the experiment was a linear arrangement of 6 router nodes, as shown in Figure 8d. This limited the candidates for parent nodes to those among the neighboring router nodes.

In the first experiment, we measured the number of DIS messages transmitted from the mobile node to select a parent node. The number of DIS message transmissions is related to the energy consumption in the mobile node. This is because even if a single DIS message is transmitted, the mobile node receives multiple DIO messages from each neighboring router node. Therefore, the number of DIS message transmissions with respect to time using each algorithm was obtained and compared.

In the second experiment, the packet loss rate of the transmissions from the mobile node was obtained. The packet loss can happen as a mobile node moves to a location that is not covered by the current parent node of the mobile node. Then, the mobile node cannot transmit a DIS message to a new parent node because the predefined time interval on a previous parent node is not expired yet. When a mobile node makes a movement, the optimal parent node for the mobile node at the new location must be selected. However, owing to the interval time defined by each algorithm, there are cases where the parent node is not selected in a timely manner. Moreover, there are cases where the mobile node escapes the transmission range of the previous parent node. If the parent node information possessed by the mobile node is not renewed at this point, losses may occur in the packet transmitted from the mobile node. Therefore, for each algorithm, the loss rate of the packets transmitted from the mobile node with respect to time was obtained and compared.

In the last experiment, the energy consumed by the mobile node in selecting a parent node was measured. Since mobile devices in most practical environments operate on batteries, the lifetime of a device is increased by reducing energy consumption. In this paper, the mobile node is also assumed to be a device operating on a battery. Energy consumption was measured based on the number of transmissions and receptions of messages used to select a parent node. The energy model for the measurement of energy consumption was based on Equation (8) in Section 4.2, and the value $d_{0}$ was set to 80% of the transmission range of the mobile node. Here, the $d_{0}$ means a distance from mobile node until a location that the signal strength on RSSI is sharply decreased [23].

### 4.2. Energy Model for Simulation

The energy consumption of data communication for the proposed algorithm is considered to be a popularly adopted energy model in previous related works [24]. In this paper, we assume an energy model where the radio dissipates $e_{elec}$ to run the transmitter or receiver circuitry, $\varepsilon_{fs}$ and $\varepsilon_{mp}$ for the transmitter amplifier depending on the distance of $d_{0}$. Here, $d_{0}$ is a distance consuming energy differently depending on the transmission and reception distance of radio frequency; thus, to transmit an *m*-bit message a distance *d* using the communication energy consumption model in each time interval of the mobile node. More details are represented in Equation (8).

(8) $$E\left({MobileNode}_{n}\right)=\left\{\begin{array}{c} 2\left(e_{elec}\left(m\right)+\varepsilon_{fs}d^{2}m\right)+e_{elec}\sum_{i=1}^{n}m_{i}\;if\;d<d_{0}\\ 2\left(e_{elec}\left(m\right)+\varepsilon_{mp}d^{4}m\right)+e_{elec}\sum_{i=1}^{n}m_{i}\;if\;d\geq d_{0} \end{array}\right.$$

Equation (8) is composed of the following two components. The first one is the energy consumption value to transmit an *m*-bit message a distance *d* (one for DIS message and one for DAO message) between a mobile node and neighbor router nodes. Another component is the energy consumption value for receiving DIO messages between a mobile node and neighbor router nodes (between one and *n*). In Equation (8), the transmission range and traffic load are important factors when the energy consumption values are derived in the communication process to select a parent node. We will use the model in Section 5.

## 5. Performance Evaluations

### 5.1. Cumulative Transfer Count

**Figure 9:**
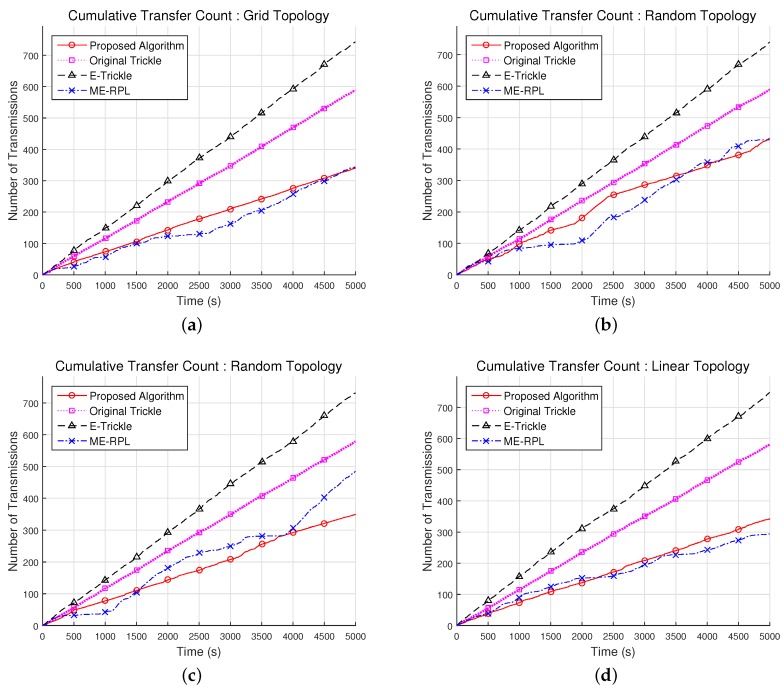
Result of cumulative transfer count with different network topologies. (**a**) Grid topology with 36 router nodes; (**b**) Random topology with 36 router nodes; (**c**) Random topology with 72 router nodes; (**d**) Linear topology with 6 router nodes.

Figure 9 shows the cumulative counts of DIS messages transmitted by the mobile node to select a parent node in the network topologies shown in Figure 8. As shown in Figure 9a, in a grid network topology consisting of 36 router nodes, the proposed algorithm exhibits a cumulative DIS message transmission count that is 40.1% and 52.7% lower than that of the original trickle algorithm and the E-trickle algorithm, respectively. However, the proposed algorithm showed an 11.6% increase over ME-RPL. As shown in Figure 9b,c, in random network topologies consisting of 36 and 72 router nodes, the proposed algorithm results in 23.3% and 37.9% fewer cumulative transmission counts than the original trickle algorithm on average, respectively; and 38.3% and 50.7% fewer cumulative transmission counts than the E-trickle algorithm. In random network topology consisting of 36 router nodes, the proposed algorithm showed a 9.1% increase as compared to ME-RPL, but it showed a decrease of 13.2% in the random network topology composed of 72 router nodes. As shown in Figure 9d, in a linear network topology consisting of 6 router nodes, the proposed algorithm has 40.3% fewer transmission counts than the original trickle algorithm on average and 53.9% fewer transmission counts than the E-trickle algorithm. However, the proposed algorithm showed a 5.0% increase over ME-RPL.

From the experimental results, it can be deduced that the proposed algorithm requires fewer DIS message transmissions for parent node selection than the original trickle algorithm or the E-trickle algorithm, but requires more than ME-RPL. In other words, the movement of a mobile node leads to a change in the network topology, and the proposed algorithm allows for the detection of this change with fewer messages than existing algorithms except ME-RPL. In the original trickle algorithm, a mobile node receives inconsistent information when the network topology is changed. From this information, the interval is set at the minimum value, and τ is selected randomly from within the range of [*I*/2, *I*]. While the E-trickle algorithm also sets the interval at the minimum value when the network topology is changed, the algorithm selects τ randomly from within the range of [0, *I*], unlike the original trickle algorithm. Owing to this difference, the E-trickle algorithm transmits more messages than the original trickle algorithm in order to enforce consistency in the network. When the mobile node moves and receives information from neighboring router nodes, ME-RPL adjusts the time interval by multiplying or dividing the constant value. By contrast, the proposed algorithm selects the interval dynamically based on the time required for the mobile node to escape the transmission range of the parent node. Hence, the proposed algorithm was capable of achieving the consistency of the network with fewer messages than the original trickle and E-trickle algorithms. However, since ME-RPL has the property of performing the division operation when topology is changed, it maintains network consistency with fewer messages than the proposed algorithm.

### 5.2. Packet Loss Rate

#### 5.2.1. Packet Loss Rate with Different Network Topologies

**Figure 10:**
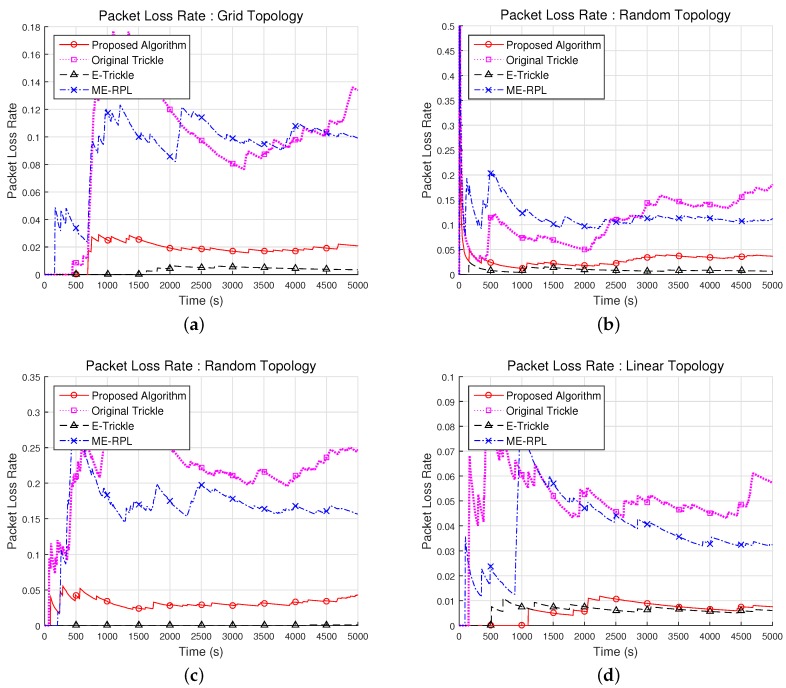
Result of packet loss rate with different network topologies. (**a**) Grid topology with 36 router nodes; (**b**) Random topology with 36 router nodes; (**c**) Random topology with 72 router nodes; (**d**) Linear topology with 6 router nodes.

Figure 10 shows the loss rates of the packets from the mobile node in the network topologies shown in Figure 8. In the 36-node grid network topology, the average packet loss rates of the proposed algorithm, the original trickle algorithm, the E-trickle and the ME-RPL algorithm were found to be 1.7%, 9.6%, 0.3% and 9.1%, respectively, as shown in Figure 10a. Therefore, while the proposed algorithm has 82.2% and 81.1% lower packet loss rate than the original trickle algorithm and the ME-RPL on average, the E-trickle algorithm was 81.7% lower than the proposed algorithm.

In the two random network topologies consisting of 36 and 72 router nodes, the average packet loss rates were found to be 3.0% and 3.1% for the proposed algorithm, 10.9% and 22.2% for the original trickle algorithm, 0.9% and 0.01% for the E-trickle algorithm and 11.9% and 16.6% for the ME-RPL, respectively. This is shown in Figure 10b,c. Therefore, the average packet loss rates of the proposed algorithm were 72.7% and 85.9% lower than those of the original trickle algorithm; the average packet loss rates of the proposed algorithm were 74.9% and 81.2% lower than those of the ME-RPL; and the average packet loss rates of the E-trickle algorithm were 70.5% and 99.6% lower than those of the proposed algorithm. From the results shown in Figure 10b, it can be observed that the standard deviations are 2.1%, 4.7%, 0.3%, and 3.1% for the proposed algorithm, the original trickle algorithm, the E-trickle and the ME-RPL algorithm, respectively. From Figure 10c, it can be seen that the standard deviations are 0.7%, 5.1%, 0.03%, and 4.6% for the proposed algorithm, the original trickle algorithm, the E-trickle algorithm and the ME-RPL, respectively.

As shown in Figure 10d, the average packet loss rates in the 6-node linear network topology were found to be 0.6%, 5.1%, 0.6% and 3.8% for the proposed algorithm, the original trickle algorithm, the E-trickle algorithm, and the ME-RPL, respectively. Therefore, the average packet loss rates of the proposed algorithm were 88.5%, 2.9% and 84.6% lower than the original trickle algorithm, the E-trickle algorithm and the ME-RPL, respectively.

From the experimental results, it can be deduced that the packet loss rate of the packets from the mobile node is lower for the proposed algorithm than the original trickle algorithm and the ME-RPL. However, the packet loss rate of the proposed algorithm is higher than that of the E-trickle algorithm. The original trickle algorithm and the E-trickle algorithm differ in their methods of allocating the redundancy constant *k* in addition to the results mentioned in Section 5.1. While the original trickle algorithm defines the *k*-value to be a constant, the E-trickle algorithm adjusts the *k*-value depending on the interval. Based on this difference, it can be observed from Figure 10 that the E-trickle algorithm is more advantageous than the original trickle algorithm in maintaining a consistent network in an environment where the network topology changes frequently.

Unlike the original trickle algorithm and E-trickle algorithms, the proposed algorithm and the ME-RPL do not consider the k-value in searching for the highest OF value, but collects the information of neighboring router nodes. Nevertheless, the proposed algorithm clearly shows lower packet loss rates than the original trickle algorithm and the ME-RPL and an equal or slightly higher packet loss rate than the E-trickle algorithm. The difference in results for the E-trickle algorithm is also closely related to the results in Section 5.1. If a mobile node transmits DIS messages more frequently per unit time, the neighboring router nodes also respond more frequently, which reduces the packet loss rate. While the proposed algorithm maintains network consistency using fewer messages than the E-trickle algorithm, it cannot accurately match the time interval for parent node selection owing to the random speed and direction of motion of the mobile node. Hence, the proposed algorithm exhibits an equal or slightly higher packet loss rate than the E-trickle algorithm.

The results in Figure 10 varied more significantly in random network topologies than in network topologies with a certain pattern. However, as shown in Figure 10b,c, a change in the number of router nodes in a random network topology did not lead to a significant variation in the results. Therefore, it was hypothesized that the proposed and existing algorithms are affected more by the speed of the mobile node than the number of router nodes. An experiment was conducted to test this hypothesis.

#### 5.2.2. Packet Loss Rate with Varying Mobile Node Speed

**Figure 11:**
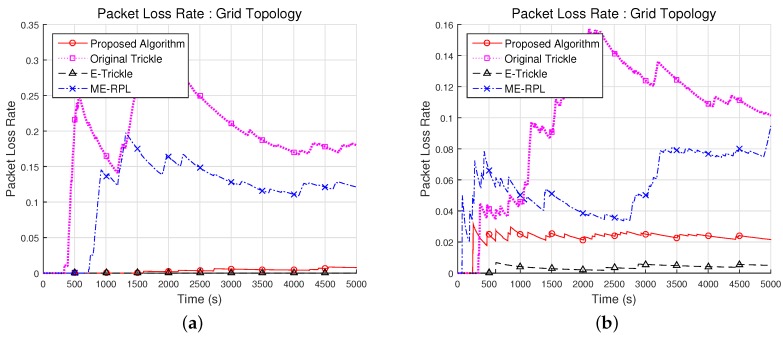
Result of packet loss rate with varying mobile node speed in grid network topology. (**a**) Packet loss rate with 1.25 m/s velocity; (**b**) Packet loss rate with 2.5 m/s velocity.

Figure 11 shows the relationship between the packet loss rate and the speed of motion of a mobile node in network topologies shown in Figure 8a. The experiment considered a situation where the mobile node moves in random directions at constant speeds, which were fixed at 1.25 m/s and 2.5 m/s, respectively.

In the grid network topology, the average packet loss rates with a mobile node speed of 1.25 m/s were 0.3%, 19.4%, 0% and 11.3% for the proposed algorithm, the original trickle algorithm, the E-trickle algorithm and the ME-RPL, respectively, as shown in Figure 11a. Therefore, the average packet loss rates were 98.3% and 97.1% lower in the proposed algorithm than in the original trickle algorithm and the ME-RPL, and the average packet loss rate of the proposed algorithm was higher than that of the E-trickle algorithm. From the results in Figure 11a, standard deviations of 0.3%, 7.7% and 5.4% were observed for the proposed algorithm, the original trickle algorithm and the ME-RPL, respectively.

When the mobile node speed was set at 2.5 m/s in the grid network topology, the average packet loss rates of the proposed algorithm, the original trickle algorithm, the E-trickle algorithm and the ME-RPL were 2.3%, 10.0%, 5.7% and 0.4%, respectively. Therefore, the average packet loss rates of the proposed algorithm were 77.2% and 60.4% lower than that of the original trickle algorithm and the ME-RPL, and the average average packet loss rate of the E-trickle algorithm was 84.4% lower than that of the proposed algorithm. In the results shown in Figure 11b, standard deviations of the proposed algorithm, the original trickle algorithm, the E-trickle algorithm and the ME-RPL were 0.5%, 4.2%, 0.2% and 1.9%, respectively.

From the experimental results, it can be inferred that the packet loss rate of the proposed algorithm with different mobile node speeds is lower than that of the original trickle algorithm and the ME-RPL, while it is higher than that of the E-trickle algorithm. With a lower mobile node speed, there are fewer changes in the parent node and the neighboring router nodes. Owing to fewer changes in the parent node, the network topology does not vary frequently either.

However, the original trickle algorithm and the ME-RPL increase the time interval as the network stabilizes. At this time, when the node moves, a packet loss occurs because it cannot transmit a message to find the neighboring router nodes. The E-trickle algorithm also increases the time interval as the network becomes stable. However, the E-trickle algorithm lowers the packet loss rate because the mobile node initializes the time interval for finding the parent when it receives an inconsistent message from the neighboring node. Conversely, if the mobile node speed is high, the network topology changes frequently. At this time, the time interval for finding the parent is shorter than when the network topology is stabilized. Therefore, the packet loss rate is lower than when the mobile node is slow.

As shown in Figure 11a,b, the proposed algorithm and the E-trickle algorithm showed better performance than the original trickle algorithm and the ME-RPL. The proposed algorithm has better performance than the original trickle algorithm and the ME-RPL, which update the time interval by calculating the constant value, although the performance is lower than the E-trickle algorithm, which continuously updates the time interval. In addition, the proposed algorithm increases the packet loss rate as the mobile node speed increases because the time interval for selecting the parent node does not exactly match due to the random movement characteristics of the mobile node, as mentioned in Section 5.2.1.

### 5.3. Energy Consumption of the Mobile Node

**Figure 12:**
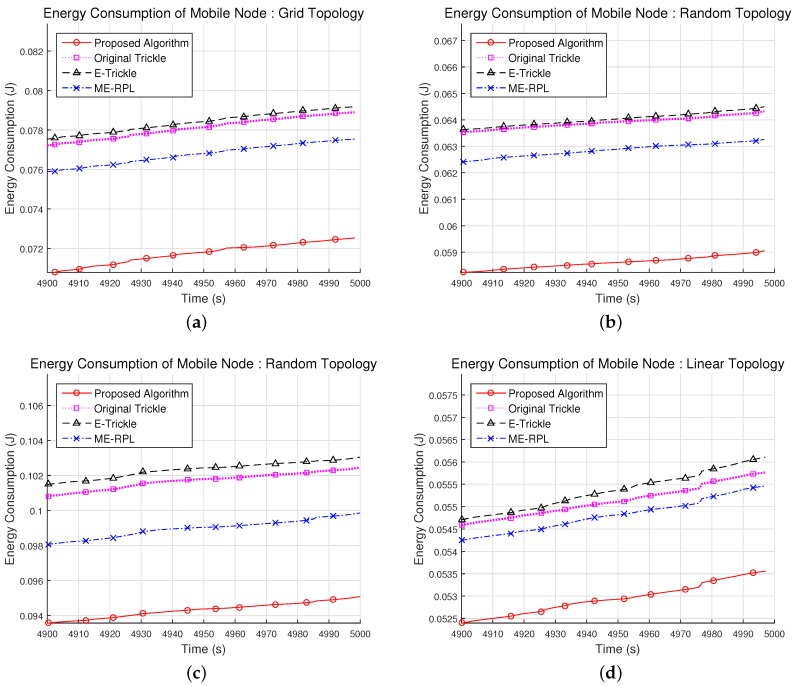
Result of energy consumption in a mobile node with different network topologies. (**a**) Grid topology with 36 router nodes; (**b**) Random topology with 36 router nodes; (**c**) Random topology with 72 router nodes; (**d**) Linear topology with 6 router nodes.

Figure 12 shows the cumulative energy consumption expended by a mobile node for parent node selection in the network topologies shown in Figure 8. The measured energy consumption of the mobile node includes the energy used during the transmission and reception of DIS, DIO and DAO messages. In the 36-node grid network topology, the total energy consumption of the mobile node was 72.5 mJ, 78.9 mJ, 79.2 mJ and 77.5 mJ for the proposed algorithm, the original trickle algorithm, the E-trickle algorithm and the ME-RPL, respectively, as shown in Figure 12a. Therefore, the energy consumption of the proposed algorithm was 8.1% and 8.4% lower than that of the original trickle algorithm and the E-trickle algorithm and 6.5% less than that of the ME-RPL.

In the 36- and 72-node random network topologies, the total energy consumption of the mobile node was 59.1 mJ and 95.1 mJ, respectively, for the proposed algorithm; 64.3 mJ and 102.4 mJ for the original trickle algorithm; 63.3 mJ and 99.9 mJ for the ME-RPL; and 64.5 mJ and 103.0 mJ for the E-trickle algorithm; as shown in Figure 12b,c. Therefore, the total energy consumption of the proposed algorithm was 8.2% and 7.1% lower than that of the original trickle algorithm, 8.4% and 7.7% lower than that of the E-trickle algorithm on average, respectively, and 6.7% and 4.8% lower than that of the ME-RPL.

As shown in Figure 12d, in the 6-node linear network topology, the total energy consumption of the mobile node was 53.6 mJ for the proposed algorithm, 55.8 mJ for the original trickle algorithm, 56.1 mJ for the E-trickle algorithm and 55.5 mJ for the ME-RPL. Therefore, the energy consumption of the proposed algorithm was 3.9% and 4.5% lower than that of the original trickle algorithm and the E-trickle algorithm on average and 3.4% less than that of the ME-RPL.

From the experimental results, it can be deduced that the energy consumed by the mobile node for parent node selection is lower for the proposed algorithm than for the original trickle, the E-trickle algorithm or the ME-RPL. Fundamentally, energy consumption is proportional to the number of messages transmitted and received by the mobile node for the selection of a parent node. However, from the results shown in Figure 12a,b, it can be seen that the original trickle algorithm consumed energy similar to the E-trickle algorithm, despite the fact that the E-trickle algorithm transmitted more messages than the original trickle algorithm, as shown in Figure 9. Similarly, the ME-RPL, which has a smaller number of sending/receiving messages in Figure 9, also consumes relatively high energy.

Based on this observation, one can reach the conclusion that the original trickle algorithm and the ME-RPL exchanged more messages with the router nodes placed further than $d_{0}$ than did the E-trickle algorithm. This is closely related to the time interval for finding the parent node, and the time interval is also related to the packet loss rate. As a result, it can be seen from the experiment result that it is necessary to find a new parent node when it is relatively far from the parent node or when communication with the existing parent node is not possible. By contrast, the proposed algorithm consumed less energy for parent node selection than the existing algorithms in all situations, as shown in Figure 12. This is possible only because the proposed algorithm had a significantly lower number of messages transmitted and received for parent node selection than the previous algorithms, owing to its characteristics.

## 6. Conclusions

In this paper, we proposed an energy-efficient node mobility algorithm through timely transmission of solicitation in the RPL network. Furthermore, we analyzed the proposed algorithm through a comparative experiment with existing algorithms. To relocate a mobile node and select a new parent node, the proposed algorithm required fewer messages for selecting a new parent node than the existing algorithms. However, due to the random speed and direction of a mobile node, packet loss occurred because the mobile node could not accurately match the time interval for selecting the parent node. Nevertheless, the result showed that the proposed algorithm has better performance than the existing algorithms.

The nodes that comprise an LLN must consume energy in an efficient manner. Energy consumption is closely related to the lifetime of LLN-based services. The problem of energy consumption is an important issue that must be resolved for most mobile nodes that operate on batteries. The proposed algorithm is more energy efficient than the existing algorithms because it uses fewer messages to select the parent node than the existing algorithms. Moreover, the proposed algorithm selects a parent using a dynamic time interval that varies with the speed and direction of motion of mobile node. This reduces the time spent by the mobile node in selecting a new parent node at its new location after movement. If the time required for parent node selection is reduced, the duration of network participation of the mobile node increases correspondingly, allowing for a more stable service.

In the presented algorithm, when the time interval for a new parent selection expires during the movement of a mobile node, the mobile node should be located on the boundary of distance that the packets of the parent node can reach. However, as the presented algorithm is applied to a real environment with a variety of factors such as the number of subscribers per router, frequency interference/reflection/attenuation, ping-pong effect, near/far problem, and so on, the mobile node may be not located on the boundary. Therefore, we will improve the proposed energy-efficient node mobility algorithm to find the exact time interval considering real environmental factors in future work. Moreover, we plan to extend our research from LLN environments to LPWA (Low Power Wide Area) environments, focusing on stable and energy-efficient methods for supporting mobile node-based services.

## Figures and Tables

**Table 1:** Simulation parameters.

Parameters	Definition
Area	100 m × 100 m, 100 m × 40 m
Simulation time	5000 s
# of router nodes	6 (linear), 36 (grid, random), 72 (random)
Transmission range	20 m
$d_{0}$	16 m
Mobility model	Random waypoint model
Mobile node speed	1.25 m/s∼2.5 m/s
$e_{elec}$	50 nJ/bit
$\varepsilon_{fs}$	10 pJ/bit/m${}^{4}$
$\varepsilon_{mp}$	0.0013 pJ/bit/m${}^{2}$

**Table 2:** Definition of the parameters in the energy model.

Parameters	Definition
*E*	Total energy consumption when *m* bits packet delivered from source to destination
$e_{elec}$	Energy dissipation of the encoding and decoding
$\varepsilon_{fs}\;\varepsilon_{mp}$	Parameters of transmitter amplifier
